# Inhibiting PI3K–AKT–mTOR Signaling in Multiple Myeloma-Associated Mesenchymal Stem Cells Impedes the Proliferation of Multiple Myeloma Cells

**DOI:** 10.3389/fonc.2022.874325

**Published:** 2022-06-20

**Authors:** Luca Heinemann, Klara Maria Möllers, Helal Mohammed Mohammed Ahmed, Lanying Wei, Kaiyan Sun, Subbaiah Chary Nimmagadda, Daria Frank, Anja Baumann, Alexandra M. Poos, Martin Dugas, Julian Varghese, Marc-Steffen Raab, Cyrus Khandanpour

**Affiliations:** ^1^ Medical Department A, University Hospital Münster, Münster, Germany; ^2^ Institute of Medical Informatics, University of Münster, Münster, Germany; ^3^ Clinical Cooperation Unit (CCU) Molecular Hematology/Oncology, German Cancer Research Center (DKFZ), Heidelberg, Germany; ^4^ Department of Internal Medicine V, Heidelberg University Hospital, Heidelberg, Germany; ^5^ Institute of Medical Informatics, Heidelberg University Hospital, Heidelberg, Germany; ^6^ Department of Haematology and Oncology, University Hospital of Schleswig-Holstein, Lübeck, Germany

**Keywords:** multiple myeloma, PI3K–AKT–mTOR signaling, mesenchymal stem cells, pictilisib, bone marrow niche

## Abstract

The microenvironment of cancer cells is receiving increasing attention as an important factor influencing the progression and prognosis of tumor diseases. In multiple myeloma (MM), a hematological cancer of plasma cells, mesenchymal stem cells (MSCs) represent an integral part of the bone marrow niche and tumor microenvironment. It has been described that MM cells alter MSCs in a way that MM-associated MSCs promote the proliferation and survival of MM cells. Yet, our understanding of the molecular mechanisms governing the interaction between MM cells and MSCs and whether this can be targeted for therapeutic interventions is limited. To identify potential molecular targets, we examined MSCs by RNA sequencing and Western blot analysis. We report that MSCs from MM patients with active disease (MM-Act-MSCs) show a distinct gene expression profile as compared with MSCs from patients with other (non-) malignant diseases (CTR-MSCs). Of note, we detected a significant enrichment of the PI3K–AKT–mTOR hallmark gene set in MM-Act-MSCs and further confirmed the increased levels of related proteins in these MSCs. Pictilisib, a pan-PI3K inhibitor, selectively reduced the proliferation of MM-Act-MSCs as compared with CTR-MSCs. Furthermore, pictilisib treatment impaired the MM-promoting function of MM-Act-MSCs. Our data thus provide a deeper insight into the molecular signature and function of MSCs associated with MM and show that targeting PI3K–AKT–mTOR signaling in MSCs may represent an additional therapeutic pathway in the treatment of MM patients.

## 1 Introduction

Multiple myeloma (MM) is a hematologic malignancy characterized by the proliferation of a plasma cell clone within the bone marrow (BM). The disease causes osteolytic lesions, anemia, and secondary immunodeficiency by repression of the physiological BM (1). Mesenchymal stem cells (MSCs) represent an essential cellular component of the non-hematopoietic BM microenvironment which interacts with aberrant plasma cells (2). Their interplay is mediated by cytokines such as IL-6 (3) or by direct cell–cell interaction through adhesion molecules such as VLA-4 and VCAM-1 (4, 5), thus resulting in increased proliferation and survival of MM cells, drug resistance, and angiogenesis (3, 6). Various studies assessed the function of MSCs in hematological diseases such as acute myeloid leukemia, myelodysplastic syndrome, or MM at the functional and genomic levels (7–11). We were therefore interested in the molecular mechanisms by which MM-associated MSCs influence MM cells and whether the identified signaling nodes could be targeted on a therapeutic level.

In this study, we compared MSCs obtained from donors with active MM disease (MM-Act-MSCs), in remission (MM-Rm-MSCs), and MSCs from donors with other malignant/non-malignant diseases (CTR-MSCs). Previous studies have shown that MM-Act-MSCs supported the proliferation of MM cells more than CTR-MSCs (5). Although these tumor-promoting effects were known early, the molecular background for this distinctive feature remains less well understood.

We, therefore, investigated the transcriptome of MM-Act- and CTR-MSCs to identify abnormal signaling and evaluated whether these changes induce proteomic alterations affecting MM cell proliferation and survival. Among others, we detected a significant enrichment of the PI3K–AKT–mTOR hallmark gene set. We were interested in the PI3K signaling pathway since it is one of the most frequently activated pathways in malignant cells (12, 13), including MM cells (14). It regulates essential processes such as cell proliferation, survival, migration, and cell metabolism. The activation of PI3K catalyzes the formation of the secondary messenger phosphatidylinositol-3,4,5-bisphosphonate. This substance acts as a membrane anchor and recruits target proteins such as AKT at the cell membrane and activates them through phosphorylation. One central downstream target activated by AKT is mTOR, an important regulator of cell proliferation (15, 16).

Finally, we evaluated whether targeting the PI3K–AKT–mTOR signaling by the pan-PI3K inhibitor pictilisib could impede the MM-supporting function of MM-associated MSCs.

## 2 Methods

### 2.1 Sample Acquisition and Cell Culture

BM aspirates were acquired from patients (**Supplementary Table 1**) as part of a study at the University Hospital Münster. The study was approved by the Institutional Review Board of the University of Muenster and the Westphalian Physician Association (2018-452-f-S). All samples were obtained after informed consent and in accordance with the Declaration of Helsinki. Mononuclear cells were isolated by density gradient centrifugation with Pancoll human (PAN Biotech, Germany) and cultured in Dulbecco’s modified eagle medium (DMEM + GlutaMAX, Gibco, Thermo Fisher Scientific, MA, USA) supplemented with 10% fetal calf serum (FCS, Sera Plus, PAN Biotech) and 3 ng/ml recombinant human fibroblast growth factor (Preprotech, Germany). MSCs between passages 3 and 7 were included in the experiments. The MM cell lines used were a kind gift from Prof. Michael Hummel (Charité – University Medicine, Berlin, Germany), Dr. Toril Holien (Norwegian University of Science and Technology, Trondheim, Norway), and Prof. Stephan Mathas (Max Delbrück Center, Berlin, Germany) and were cultured in Roswell Park Memorial Institute 1640 medium (Gibco, Thermo Fisher Scientific) + 20% FCS (MM.1S, SKMM-2, U-266, OPM-2, LP-1) or Iscove’s modified Dulbecco’s medium (IMDM, Gibco, Thermo Fisher Scientific) + 20% human plasma + 20 U/ml heparin (Sigma Aldrich, MO, USA) + 50 µM β-mercaptoethanol (PAN Biotech) supplemented with 10 ng/ml recombinant human IL-6 (Preprotech) (KJON). MM cells were subcultured every 3 days and 1 day before an experiment.

### 2.2 Cell Characterization

MSCs were subcultured at 80% confluence and screened by flow cytometry for MSC typical anti-human surface markers against CD14, CD31, CD34, CD45, and CD138 as negative markers and CD90, CD105, and CD73 as positive markers for MSCs (**Supplementary Table 2**). After staining, characterization was performed using the Attune™ NxT Flow Cytometer (Thermo Fisher Scientific). Compensation adjustments were set according to Attune™ Acoustic Focusing Cytometer instructions (17). Results were analyzed using FlowJo™ v10.8 (BD Biosciences, NJ, USA) (**Supplementary Figure 1**).

### 2.3 Cell Doubling Time

MSCs (*n* > 6 per group) were cultured, counted, and reseeded at 80% confluency. Samples requiring more than 10 days to reach 80% confluency were excluded. Doubling time was calculated using the following formula (18, 19):


$$doubling\;time=\frac{t*\log\left(2\right)}{\log\left(N_{2}\right)-\log\left(N_{1}\right)}$$

*N*_1_: cell number at seed

*N*_2_: cell number after harvest

*t*: time difference between passages 1 and 2 [h]

### 2.4 Assays to Measure the Influence of MSC and MM Cell Coculture

#### 2.4.1 MM Cell Proliferation Assay

In a 12-well plate, 1 × 10^4^ MSCs/well (*n* = 3 per group) were seeded and allowed to adhere to the plate overnight. On the following day, an MM cell suspension (1 × 10^5^ MM cells in 1 ml IMDM + 20% FCS) was either filled into an empty well (monoculture) or an MSC-plated well (coculture). After 3 days, 1 ml of fresh media was added to each well onto the old media. After 6 days, the proliferation of MM cells was measured using trypan blue staining assay.

#### 2.4.2 Transwell Proliferation Assay

Initially, 5 × 10^3^ MSCs/well were seeded into a 24-well plate (Costar™ Transwell™ Permeable Support 8.0 µm Polycarbonate Membrane, Corning Inc., NY, USA) and allowed to adhere to the plate overnight. On the following day, an MM cell suspension (5 × 10^4^ MM cells in 0.5 ml IMDM + 20% FCS) was either filled into an MSC-free well (monoculture) or an MSC-plated well with and without a Transwell insert (coculture with and without direct cell–cell contact). Furthermore, 5 × 10^4^ MM cells were seeded into 0.5 ml MSC-preconditioned IMDM + 20% FCS (monoculture in supernatant). After 3 days, the MM cells were quantified using trypan blue staining assay.

#### 2.4.3 MSC Proliferation Assay

In a 6-well plate, 2 × 10^4^ MSCs/well (*n* = 3 per group) were seeded and allowed to adhere to the plate overnight. On the following day, the seeding media was replaced with 2 ml IMDM + 20% FCS or an MM cell suspension (2 × 10^5^ MM cells in 2 ml IMDM + 20% FCS). After 3 days, the cells were harvested, counted, and stained with CD90 according to the manufacturer’s instructions. Results were analyzed using FlowJo™ v10.8.

### 2.5 RNA Sequencing

RNA sequencing data of MM-Act-MSCs (*n* = 6) and CTR-MSCs (*n* = 5) were compared to detect differentially expressed genes. We obtained the samples from the primary culture and analyzed them by flow cytometry, and after a quality control performed with the 4200 TapeStation (Agilent, CA, USA) in the Sample Processing Lab (German Cancer Research Center, Heidelberg, Germany), we included them in the study. RNA was extracted using the AllPrep DNA/RNA/miRNA Universal Kit (Qiagen, Netherlands). Following the TrueSeq stranded protocol (Illumina, CA, USA), we sequenced RNA in a 100-bp paired-end run on HiSeq 4000 (Illumina). Transcript quantification was performed using Salmon (Robert Patro, Stony Brook University) by quasi-mapping against the human reference transcriptome (GRCh38, cDNA). Normalization of the read counts and differential expression analysis were performed with DESeq2 (Michael Love, Harvard School of Public Health). Gene set enrichment analysis was realized by GSEA software (Broad Institute Inc., MA, USA) based on MSigDB hallmark gene sets. The raw and processed RNA sequencing data are available through the GEO series accession number GSE196297.

### 2.6 Western Blot Analysis

Cell extracts were produced by reducing and denaturing 2 × 10^4^ MSCs/well in electrophoresis buffer (NUPAGE^®^ LDS Sample Buffer/Reducing Agent, Invitrogen, MA, USA). The samples were separated by a 10% polyacrylamide gel at 100 V for 90 min at RT and blotted onto a polyvinylidene difluoride membrane (Immobilon E, 0.45 µM, Merck Millipore, MA, USA) at 100 V for 90 min at 4°C. The membrane was blocked in TBS solution containing 0.1% Tween 20 (Sigma Aldrich) and 5% non-fat dry milk for 75 min at RT and incubated in primary antibodies against PI3K-α, PI3K-β, AKT, p-AKT, mTOR, p-mTOR, and β-actin at 4°C overnight (**Supplementary Table 3**). After washing, the membranes were incubated in horseradish peroxidase-conjugated secondary antibody solution in 5% milk in TBS-T at RT for 2 h. After another washing step, Radiance Plus (Azure Biosystems, CA, USA) was used as the detection reagent kit. Results of *n >*3 per donor group were analyzed by ImageJ (National Institutes of Health, MD, USA). Normalization was performed to the internal loading control β-actin.

### 2.7 Flow Cytometry Analysis for p-S6

In a 6-well plate, 5 × 10^4^ MSCs/well (*n* = 3 per group) were seeded and allowed to adhere to the plate overnight. On the following day, the seeding media and the non-adherent cells within were replaced with IMDM + 20% FCS with or without 2 × 10^5^ MM.1S cells. After 3 days, the cells were harvested, stained with CD90, permeabilized with Cytofix/Cytoperm (BD Biosciences), and stained with p-S6 according to the manufacturer’s instructions. Results were analyzed using FlowJo™ v10.8.

### 2.8 Assays to Measure the Influence of Pictilisib on MSCs and MM Cell Lines

#### 2.8.1 Seeding

In a 12-well plate, 1 × 10^4^ MSCs/well (*n* = 3 per group) were seeded and allowed to adhere to the plate overnight. On the following day, the medium was replaced with 1 ml IMDM + 20% FCS with a molar concentration of pictilisib (Selleck Chemicals, TX, USA) of 0.8 µM or an equivalent volume of dimethyl sulfoxide (DMSO, PAN Biotech) as carrier substance and 1 × 10^5^ MM cells (MM.1S, KJON, SKMM-2). Also, 1 × 10^5^ MM cells were seeded and treated in monoculture as control. To examine MSCs in monoculture, 5 × 10^5^ MSCs were seeded in T25 flasks and treated after adherence as described above. The cells were cultured for 3 days and analyzed for cell count and apoptosis.

#### 2.8.2 Proliferation Assay

After 3 days, MM cells were quantified by trypan blue staining assay. To quantify monocultured MSCs, they were detached with trypsin–EDTA solution 0.25% (Sigma Aldrich) for 4 min before staining and counting.

#### 2.8.3 Apoptosis Assay

The extent of apoptosis of MSCs and MM cells after pictilisib treatment at 0.8 µM was analyzed by flow cytometry using Annexin-V (APC, anti-human, BioLegend, CA, USA) to detect early apoptotic cells and propidium iodide (PI, Thermo Fisher Scientific) to stain late apoptotic cells. Non-cellular debris was excluded, and Annexin^−^/PI^−^ cells were considered viable and counted.

### 2.9 Statistical Analysis

Statistical analysis was performed using GraphPad Prism version 9.1.2 for Windows (GraphPad Software Inc., CA, USA). Data are presented as mean ± SEM. Information on the statistical tests used is provided in the figure legends. A *p*-value <0.05 was considered significant.

## 3 Results

### 3.1 MM-Act-MSCs Supported the Growth of MM Cells More Than MM-Rm- and CTR-MSCs

In many hematologic malignancies, MSCs were found to be functionally altered to support tumor cell proliferation in contrast to MSCs derived from healthy donors.

**Figure 1:**
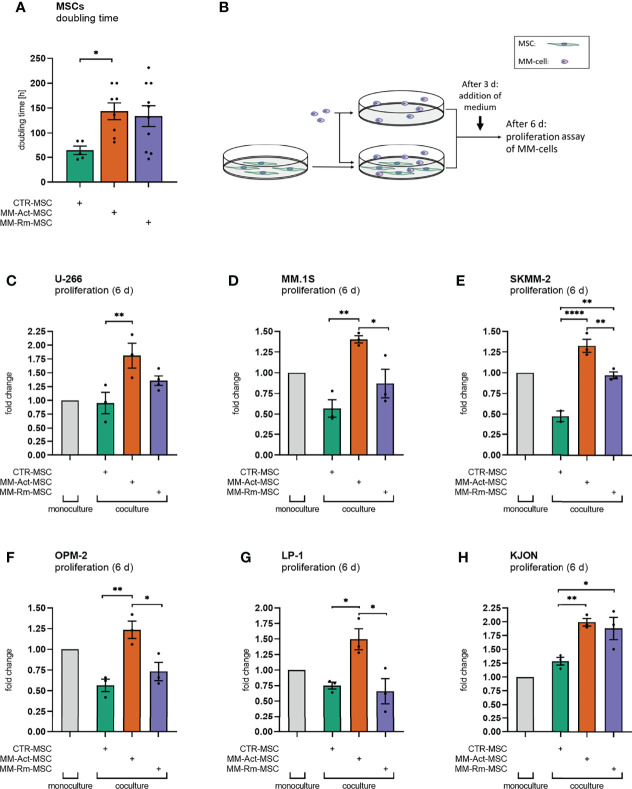
Doubling time of mesenchymal stem cells (MSCs) and proliferation assay of multiple myeloma (MM) cell lines (mono- and coculturing setup, 6 days). **(A)** Bar chart comparing the doubling time of MM-Act-MSCs, MM-Rm-MSCs, and CTR-MSCs. **(B)** Schematic overview of the experimental design of MSC and MM cell mono- and cocultures: MSCs were seeded and could attach within 1 day. MM cells were either cultured alone or together with MSCs. After 3 days of coculturing, IMDM + 20% FCS was added. After 6 days, the proliferation assay was performed. **(C–H)** Bar charts showing the fold changes of MM cell proliferation after 6 days, normalized to MM cell count in monoculture setup. Data are presented as mean ± SEM. Significance was calculated using one-way ANOVA. *p ≤ 0.05, **p ≤ 0.01, ****p ≤ 0.0001.

Therefore, we first investigated the functional differences between MSCs from MM patients with active disease and in remission and the control donors. For this purpose, we examined the proliferation capacity of the MSCs. In our cohort, BM samples from MM patients with active disease were expandable in only 48% of the cases, whereas 65% of the MM samples were in remission and 70% of the control samples proliferated well. Moreover, we observed an increased doubling time in MM-Act-MSCs compared with CTR-MSCs (**Figure 1A**, 2.3-fold, *p* < 0.05) that did not correlate with age, sex, and bone marrow infiltration (**Supplementary Figures 2A–D**). In addition, we found MM-Act- and MM-Rm-MSCs proliferating lesser in coculture with MM cells, whereas CTR-MSCs were not affected significantly (**Supplementary Figure 2E**). These findings indicate that MM cells may reduce the proliferation capacity of MM-MSCs.

We next examined the ability of MM-Act-, MM-Rm-, and CTR-MSCs to support the proliferation of various MM cell lines (U-266, MM.1S, SKMM-2, OPM-2, LP-1, KJON). Therefore, we either cultured the MM cells alone or cocultured them with MSCs as indicated (**Figure 1B**). Interestingly, the presence of MM-Act-MSCs enhanced the proliferation of MM cells as compared with MM cells cultured alone (**Figures 1C–H**). Furthermore, MM-Act-MSCs supported the growth of MM cells to a higher extent than MM-Rm- and CTR-MSCs, indicating that MSCs play a relevant role in influencing MM cell proliferation. Hence, there is a reciprocal relationship between MM cells and MSCs influencing each other’s proliferation.

To investigate whether direct cell–cell interaction is important for the MM-promoting effect of MM-Act-MSCs, we compared the MM cell proliferation in coculture with MSC in direct cell–cell contact, separated by Transwell inserts and in MSC-preconditioned supernatant (**Supplementary Figure 2F**). Notably, the proliferation of MM cells in direct contact with MM-Act-MSCs was increased to 1.4-fold compared with MM cells spatially separated from the MSCs or cultured in MSC-preconditioned supernatant (*p* < 0.01). These data point toward the essential role of direct cell–cell contact between MM-Act-MSCs and MM cells for MM cell proliferation.

In summary, we found that MM-Act-MSCs were indeed functionally altered. They proliferated less than CTR-MSCs, which was further enhanced upon direct contact with the malignant cells. At the same time, they showed a proliferation-promoting capacity for MM cells that is strongly mediated by direct cell–cell contact.

### 3.2 MM-Act-MSCs Have a Distinct Gene Expression Profile Compared With CTR-MSCs

Having found evidence for the functional differences between MM-Act- and CTR-MSCs, we next evaluated the differences between these MSC types on a transcriptomic level by performing RNA sequencing.

**Figure 2:**
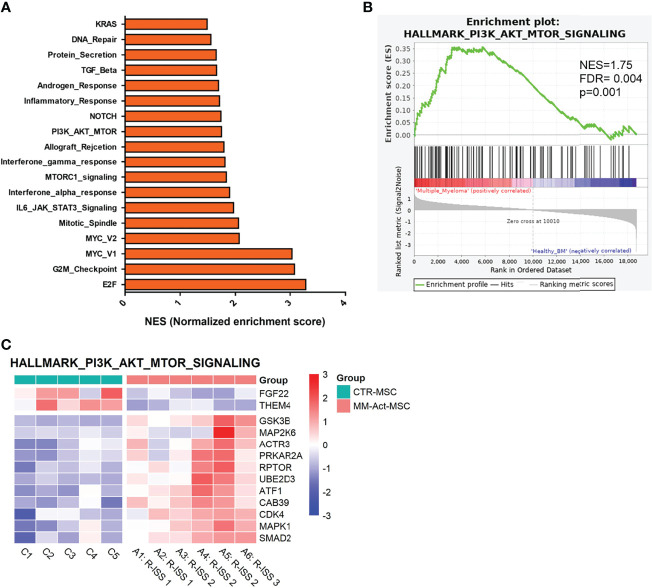
Gene enrichment plot comparing MM-Act-MSCs and CTR-MSCs in PI3K–AKT–mTOR signaling pathway. **(A)** Bar chart presenting the normalized enrichment scores of gene sets in MM-Act-MSCs compared with CTR-MSCs (adjusted *p*-value < 0.05). **(B)** Enrichment plot of the PI3K–AKT–mTOR hallmark gene set was positively enriched in MM-Act-MSCs (*n* = 6) as compared with CTR-MSCs (*n* = 5). **(C)** Heatmap of differentially expressed genes (*p* < 0.05) within the PI3K–AKT–mTOR hallmark gene set showing an enrichment in MM-Act-MSCs as compared with CTR-MSCs. NES, normalized enrichment score.

Between MM-Act-MSCs (*n* = 6) and CTR-MSCs (*n* = 5), we detected 2,174 significant differentially expressed genes (adjusted *p* < 0.05), among which 967 were upregulated in MM-Act-MSCs. We used gene set enrichment analysis on MM-Act- and CTR-MSCs to identify altered gene expression patterns. Eighteen of the 50 MSigDB hallmark gene sets were significantly enriched in MM-Act-MSCs compared with CTR-MSCs (**Figure 2A**, false discovery rate < 0.05) including PI3K–AKT–mTOR signaling (**Figures 2B, C** and **Supplementary Figure S3A**), as well as NOTCH, MYC, and inflammatory response (**Supplementary Figures 3B–D**).

### 3.3 MM-Act-MSCs Showed Enriched Levels of Proteins Involved in PI3K–AKT–mTOR Signaling Compared With CTR-MSCs

**Figure 3:**
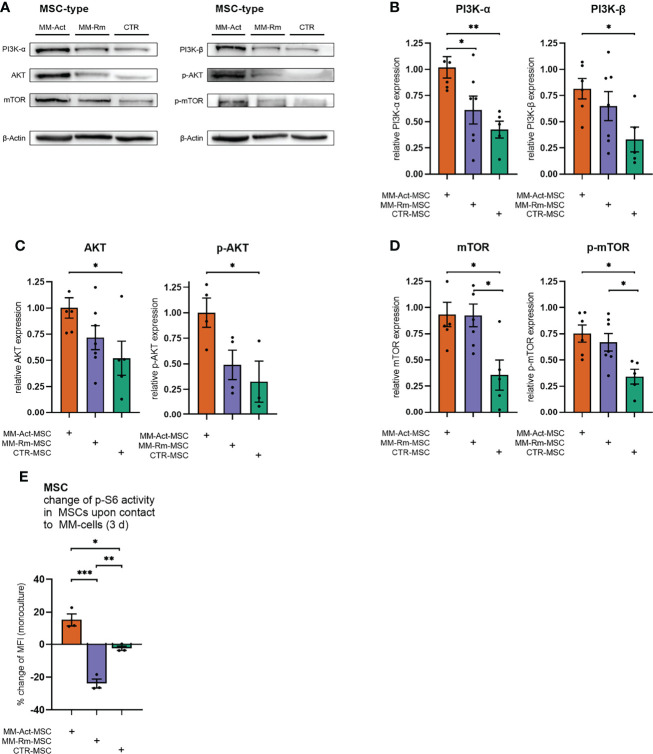
Quantification of PI3K–AKT–mTOR signaling in MM-Act-, MM-Rm-, and CTR-MSCs. **(A)** Western blot of the baseline expression of PI3K–AKT–mTOR signaling in MSCs. **(B–D)** Bar charts showing the baseline expression of PI3K–AKT–mTOR signaling in MSCs, normalized to the housekeeping protein β-actin. **(E)** Bar chart showing the percentage changes of p-S6 activity in MSCs after cocultivation with the MM cell line MM.1S for 3 days. Data are presented as mean ± SEM. Significance was calculated using one-way ANOVA. *p ≤ 0.05, **p ≤ 0.01, ***p ≤ 0.001.

Subsequently, we examined whether these transcriptomic alterations could be confirmed at the protein level (**Figures 3A–D**) by Western blot analysis.

Consistent with the transcriptome data, we found an increased level of PI3K-α in MM-Act-MSCs compared with CTR-MSCs (2.4-fold, *p* < 0.01), as well as in comparison to MM-Rm-MSCs (1.4-fold, *p* < 0.05). Additionally, we detected a significant enrichment of PI3K-β in MM-Act-MSCs compared with CTR-MSCs (2.5-fold, *p* < 0.05). One main downstream target of PI3K-α and PI3K-β is AKT. In MM-Act-MSCs, total AKT as well as T308-phosphorylated, active state AKT (p-AKT) protein levels were increased compared with CTR-MSCs (1.9-fold/3.1-fold, both *p* < 0.05). Further downstream, the protein level of mTOR, a target of AKT, was elevated and shifted to its S2448-phosphorylated active state in MM-Act-MSCs compared with CTR-MSCs (2.6-fold/2.2-fold, both *p* < 0.05). Of note, also MM-Rm-MSCs showed this enrichment and enhanced presence of the activated form of mTOR as compared with CTR-MSCs (2.6-fold/2.0-fold, both *p* < 0.05).

To determine how the activity of the PI3K–AKT–mTOR pathway changes in MSCs after contact with MM cells, we investigated the phosphorylation of ribosomal protein S6 as an indicator of mTOR activity (20) and a biomarker of stromal cell response to PI3K–AKT–mTOR inhibition (21).

After 3 days of cocultivation with the MM cell line MM.1S, we observed a significant increase in the p-S6 level of MM-Act-MSCs compared with CTR-MSCs (**Figure 3E**, *p* < 0.01), in which no relevant change in the pathway’s activity was observed. Interestingly, contact with MM cells resulted in a significant reduction of the p-S6 level in MM-Rm-MSCs.

In summary, on the RNA as well as protein level, we observed increased activity of the PI3K–AKT–mTOR pathway in MM-Act-MSCs compared with CTR-MSCs, natively and upon cocultivation with MM cells.

### 3.4 MM-Act-MSCs Mediated the Antiproliferative Effect of Pictilisib on MM Cell Proliferation

We then evaluated the functional consequences of activated PI3K–AKT–mTOR signaling. To probe this, we inhibited the PI3K–AKT–mTOR pathway in MSCs and/or MM cells by pictilisib, a potent inhibitor of all isoforms of PI3K with tolerable side effects (22). It has not only been previously tested in breast and advanced solid cancers (22, 23), but its inhibitory effect on MM cell proliferation and survival has also been investigated *in vitro* on MM cell lines (24). In a series of experiments, we determined the dose of 0.8 µM pictilisib to have a significant effect on MSCs and MM cells (**Supplementary Figures S4A, B**), and it was attainable *in vivo* (22).

**Figure 4:**
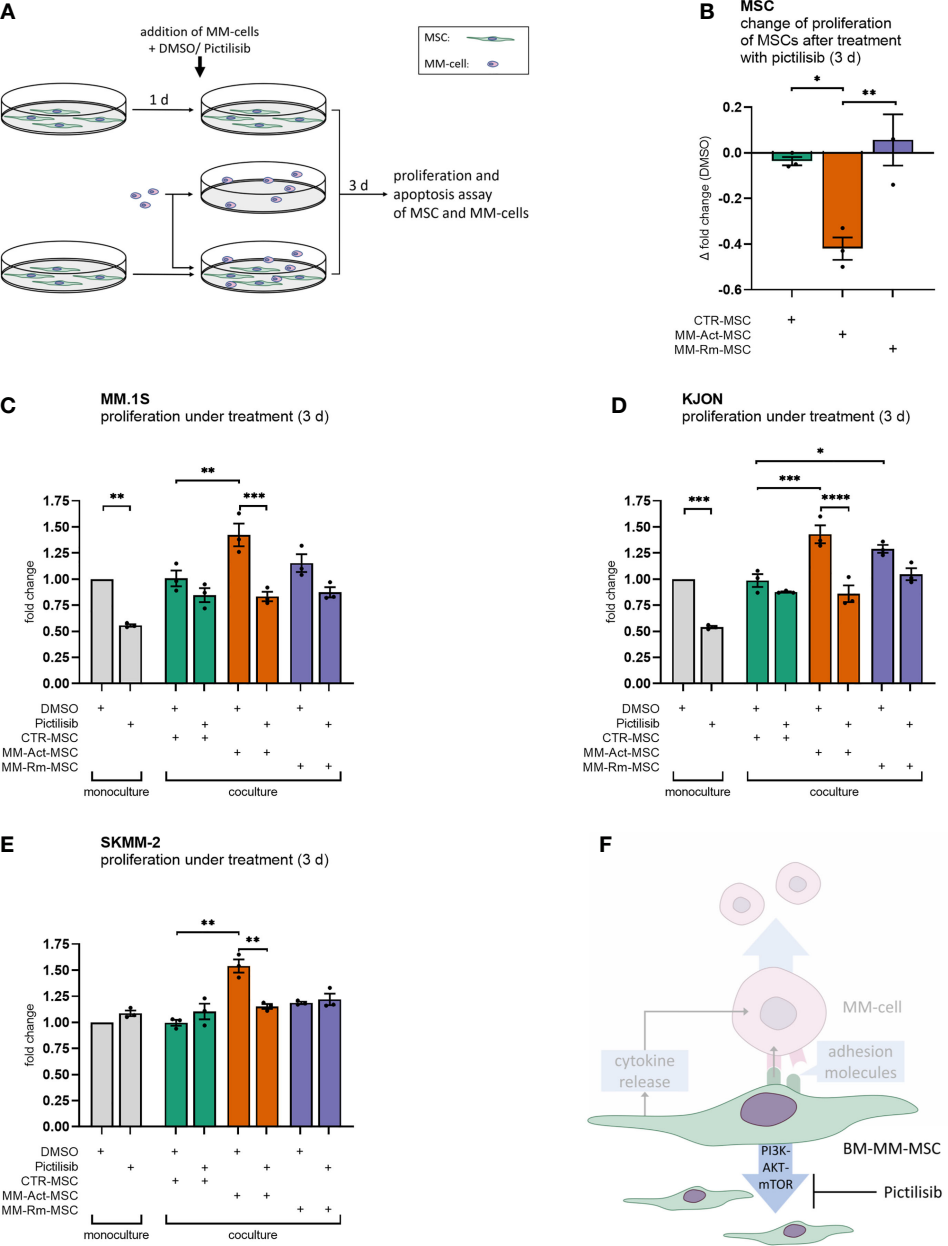
Proliferation assay of MSCs and MM cells with DMSO or pictilisib treatment (mono- and coculturing setup, 3 days). **(A)** Schematic overview of the experimental design of MSC and MM cell mono- and cocultures: MSCs were seeded and could attach within 1 day. MSCs and MM cells were cultured alone and treated with DMSO or with the pan-PI3K inhibitor pictilisib (0.8 µM). Additionally, MSCs and MM cells were cultured and treated together. After 3 days of cultivation, a proliferation assay or apoptosis assay was performed. **(B)** Bar chart showing changes in the proliferation of MSCs after 3 days of treatment with pictilisib (0.8 µM), compared with DMSO treatment. **(C–E)** Bar chart showing the fold changes of the proliferation of MM cell lines (mono- and coculturing setup) after 3 days of treatment with pictilisib (0.8 µM), normalized to cell count after DMSO treatment in MM cell monoculture. **(F)** Schematic representation of the study’s key findings: pictilisib treatment impaired the proliferation of MM-promoting MM-Act-MSCs. Thus, the treatment impeded the proliferation of MM cells when cocultured. Data are presented as mean ± SEM. Significance was calculated using one- or two-way ANOVA. *p ≤ 0.05, **p ≤ 0.01, ***p ≤ 0.001, ****p ≤ 0.0001.

We first treated the different MSC types with pictilisib (**Figure 4A**). Compared with the DMSO control, MM-Act-MSCs showed reduced proliferation by 0.4-fold. Meanwhile, pictilisib had only a marginal effect on the proliferation of MM-Rm- and CTR-MSCs (**Figure 4B**, *p* < 0.05), showing that PI3K–AKT–mTOR signaling is functionally relevant in MM-Act-MSCs.

Furthermore, we aimed to determine whether the differing effects of pictilisib on these types of MSCs also affected the growth of MM cells cocultured with them. Therefore, we set up an experiment comparing the proliferation of malignant cells either cultured alone or with MSCs while treating them with pictilisib or DMSO as a control (**Figures 4C–E**).

In monoculture, treatment of the MM cell lines MM.1S and KJON (**Figures 4C, D**) with pictilisib resulted in a proliferation reduced by 0.4-fold/0.5-fold compared with DMSO-treated cultures (*p* < 0.01). Similar to our initial findings in a coculture setting, MM-Act-MSCs enhanced the proliferation of MM.1S and KJON cells more than CTR-MSCs. However, when MM.1S and KJON cells were cocultured with MM-Act-MSCs and pictilisib was added to the culture, the proliferation supporting role of MM-Act-MSCs was completely abrogated. In the coculture with MM-Rm- and CTR-MSCs, the total extent of the antiproliferative effect of pictilisib was less pronounced.

In contrast to MM.1S and KJON cells, the cell line SKMM-2 (**Figure 4F**) was resistant to pictilisib in monoculture. Also, in the coculture with MM-Rm- and CTR-MSCs, the proliferation of SKMM-2 cells was not restricted by the treatment. Only in the coculture with the pictilisib-sensitive MM-Act-MSCs, the treatment reduced SKMM-2’s proliferation by 0.4-fold (*p* < 0.01).

We also tested whether pictilisib had proapoptotic effects on MSCs and MM cells. For this purpose, we examined the different MSC types and MM cell lines for markers of early and late apoptosis after 3 days of DMSO or pictilisib treatment. In this setup, neither the pictilisib-treated MSCs nor the treated MM cell lines showed an increased rate of apoptosis (data not shown), indicating that the effects of pictilisib are primarily antiproliferative.

## 4 Discussion

Although MSCs are currently receiving more attention in cancer research as part of the tumor microenvironment, e.g., as an essential part of an inflammatory BM niche in MM (25), many questions remain unanswered about their multifaceted and reciprocal relationship with malignant cells.

In line with the current state of research (5), we provide further evidence that MM-Act-MSCs support the proliferation of MM cells more than MM-Rm- and CTR-MSCs. In our experiments, direct cell–cell contact primarily mediated this effect.

Consistent with our gene expression profiling, previous studies showed significant differences in the transcriptome of MM-MSCs and MSCs from healthy donors (26–28) and even identified them as independent prognostic markers for the outcome and progression of MM (26) showing alterations of genes and pathways implicating cell cycling, DNA repair, inflammatory response, or differentiation processes. We observed increased RNA levels of the PI3K–AKT–mTOR signaling pathway in MM-Act-MSCs compared with CTR-MSCs. PI3K–AKT–mTOR signaling is a central pathway in the regulation of the growth of malignant and non-malignant cells (29). Furthermore, this pathway promotes an anabolic metabolism fulfilling the demands of vast proliferating cancerous cells (15).

We further detected increased levels of key proteins of the PI3K–AKT–mTOR (PI3K-α, PI3K-β, AKT, and mTOR) signaling pathway compared with CTR-MSCs. This not only could be a result of the increased RNA levels observed but also due to an enhanced protein stability or reduced degradation which our experimental setup cannot discriminate. This needs to be specified in future experiments.

In addition, increased activation of p-AKT and p-mTOR at the protein level was found in MM-Act-MSCs. Interestingly, MM-Rm-MSCs showed elevated protein levels of PI3K–AKT–mTOR signaling that was lying between MM-Act-MSCs and CTR-MSCs. For mTOR and p-mTOR, MM-Rm-MSCs showed enhanced protein levels comparable to MM-Act-MSCs. This may point toward a long-lasting alteration of MSCs after contact with malignant plasma cells.

Moreover, we showed that the activity of the PI3K–AKT–mTOR pathway, as measured by the phosphorylation of ribosomal protein S6, further increased in MM-Act-MSCs upon contact with MM cells, whereas CTR-MSCs showed no effect and MM-Rm-MSCs even responded with a downregulation. We chose a control group consisting of healthy donors and patients suffering from different malignant conditions with no detectable BM infiltration as well as patients who had previously suffered from malignant diseases but were in remission after intensive therapy and had no BM infiltration at the time of sampling. Thus, we tried to consider the impact of previous treatment and changes in the organism caused by malignant cells. Therefore, all observed effects are most likely due to direct cell–cell contact with MM cells. However, since the control group was so composed, the MSCs may have been altered by the diseases from which the patients suffered.

A targeted treatment of MM-Act-MSCs with the pan-PI3K inhibitor pictilisib led to a significant and selective restriction of the proliferation of MM-Act-MSCs, while MM-Rm- and CTR-MSCs were only marginally affected. As MM-Rm-MSCs show a comparably high level and activity of mTOR and p-mTOR in monoculture, the cause for the pictilisib insensitivity remains unclear. This needs to be investigated in future experiments.

In previous studies, PI3K inhibitors have shown potential to inhibit some MM cell lines directly (24, 30) and to overcome resistance mechanisms, e.g., against bortezomib (31). Currently, the most successful therapeutic regimens against MM utilize the simultaneous targeting of MM cells and the MM-promoting BM microenvironment by agents such as lenalidomide, thalidomide, and bortezomib (32, 33).

PI3K–AKT–mTOR signaling is constitutively activated in numerous MM cells. Therefore, some MM cell lines were described to be sensitive to pictilisib (24), which we could confirm for KJON and MM.1S. Interestingly, the antiproliferative effect of pictilisib on these sensitive MM cells was significantly different depending on whether MM cells were cocultivated with MM-Act-MSCs or MM-Rm- and CTR-MSCs. This has led us to hypothesize that beyond the direct influence of pictilisib on MM cell proliferation, the type of MSCs and their sensitivity to PI3K inhibitors contribute to the expansion of MM cells. Our observations from the SKMM-2 cell line additionally support this theory. Although SKMM-2 was pictilisib-resistant in monoculture, the drug reduced its proliferation in coculture with the pictilisib-sensitive MM-Act-MSCs. This suggests that pictilisib attenuates the proliferation-promoting capacity of MM-Act-MSCs. In line with this, the proliferation rates of SKMM-2 cocultured with MM-Rm- and CTR-MSCs remained unaffected.

In summary, pictilisib directly affected the proliferation of sensitive MM-Act-MSCs and the MM cell lines MM.1S and KJON as well as all MM cell lines cocultured with MM-Act-MSCs, regardless of whether they were primarily sensitive or resistant to the inhibitor.

Although the transcriptome changes in our RNA sequencing data (**Supplementary Table 4**) and the coculturing experiments suggest that direct cell–cell contact plays an essential role in the MM-promoting effect of MM-Act-MSCs, further studies are required to determine the precise changes in cytokine secretion and cell adhesion and whether these may be altered by pictilisib.

Previous studies have shown that MM-MSCs support MM cell survival by activation of the NF-κB, PI3K/Akt, and MAPK pathways (34). These antiapoptotic effects were mediated by both direct cell–cell contact and soluble factors inducing antiapoptotic proteins, e.g., BCL-XL, MCL1, and caspase inhibitors (4, 34, 35). We did not detect a relevant increase in the apoptosis rate in MSCs and MM cell lines by pictilisib. Hence, we assume that the antitumor effects of this drug are due to its antiproliferative effect. Thus, our data are partially in conflict with a study demonstrating an increased apoptosis rate for MM.1S by pictilisib (24). It is possible that the antiproliferative effects of pictilisib are observable at low concentrations already, but cytotoxic effects rather occur at higher concentrations.

As previously reported, pictilisib is a potent inhibitor of osteoclast differentiation and bone resorption *in vitro* and effectively prevents tumor-mediated osteolytic lesions *in vivo* by inhibition of the PI3K–AKT–GSK3β and NF-κB pathways (36). Similar results were obtained for the PI3K inhibitor BKM120 and the PI3K–mTOR inhibitor dactolisib on osteolytic bone disease in a murine MM model (37, 38). Especially for MM patients, osteolytic lesions represent a critical symptom that affects over 80% of all patients at the time of diagnosis and is associated with severe pain, pathological fractures, and the need for intensive therapy (37). This could render pictilisib a promising drug in MM treatment. However, further preclinical work is required to answer this question.

Beyond the enrichment of the PI3K–AKT–mTOR hallmark gene set, the NOTCH and MYC hallmark gene sets were found to be overexpressed. These genes and their products are known to be involved in the interaction between MSCs and malignant cells (39). Previous studies showed that NOTCH signaling causes increased release of chemokines and cytokines (40). *MYC* was detected to influence the proliferation and differentiation of MSCs (41) and to be involved in MSC-mediated drug resistance in acute myeloid leukemia (42). De Jong et al. detected an MM-specific inflammatory landscape in MSCs affecting immune modulation and tumor cell survival, and it could play a role in disease persistence (25). Also, our RNA sequencing data indicate changes in genes related to inflammation.

Considering the relevance of these pathways in MSCs for other hematological malignancies (7, 39, 43), the alterations we observed represent an additional interesting subject for future research on MM-associated MSCs. It remains to be elucidated whether genetic, epigenetic, or metabolic alterations are causative for the signaling pathways activated in MM-associated MSCs, e.g., PI3K–AKT–mTOR signaling, and how these signaling pathways are triggered by the presence of MM cells.

In summary, our study provides evidence that the PI3K–AKT–mTOR signaling pathway influences the proliferation of MM-Act-MSCs and either directly or indirectly influences the interaction between MM cells and MM-Act-MSCs. We propose that inhibiting PI3K could represent an additional approach to treat MM patients by not only targeting malignant MM cells but also dampening the supportive function of MM-Act-MSCs.

## Data Availability Statement

The datasets presented in this study can be found in online repositories. The names of the repository/repositories and accession number(s) can be found below: NCBI GEO, GSE196297.

## Ethics Statement

The study was approved by the IRB of the University of Muenster and the Westphalian Physician Association (2018-452-f-S). The patients/participants provided their written informed consent to participate in this study.

## Author Contributions

LH and KM designed the study, performed the experiments, data analysis and interpretation, and wrote the manuscript. HA designed the study and performed the experiments and data analysis. LW, AP, MD, and JV performed the data analysis and interpretation. KS, SN, DF, and CK provided essential reagents and research support. AB and M-SR performed the RNA sequencing. CK designed the study, performed the data analysis and interpretation, and wrote the manuscript. All authors contributed to the article and approved the submitted version.

## Funding

The work was supported by the Deutsche José Carreras Leukämie-Stiftung (DJCLS 17R/2018), partially by the Deutsche Krebshilfe (70112392) and Deutsche Forschungsgemeinschaft (KH331/2-3), and by the intramural funding of the Faculty of Medicine at the University Hospital of Muenster (Kha2/002/20). We acknowledge support from the Open Access Publication Fund of the University of Muenster. LH and KM were supported by the Medizinerkolleg Münster (MedK).

## Conflict of Interest

The authors declare that the research was conducted in the absence of any commercial or financial relationships that could be construed as a potential conflict of interest.

## Publisher’s Note

All claims expressed in this article are solely those of the authors and do not necessarily represent those of their affiliated organizations, or those of the publisher, the editors and the reviewers. Any product that may be evaluated in this article, or claim that may be made by its manufacturer, is not guaranteed or endorsed by the publisher.

## Supplementary Material

Supplementary Figure 1 Histogram showing an exemplary flow cytometric staining of MM-Act-MSCs in passage 4, positive for the typical MSC-surface markers as CD90, CD73, and weakly positive (in accordance with manufacturer´s instructions) for CD105 and negative for CD14, CD45, CD34, CD31 and CD138

Supplementary Figure 2
**(A)** Dot plot/Correlative analysis of MSCs´ doubling time vs. donors´ age at sampling **(B)** Box plot/Comparison of MSCs´ doubling time and donors´ sex. **(C, D)** Dot plot/Correlative analysis of MSCs´ doubling time vs. BM-infiltration in different subgroups **(E)** Bar chart showing the count of MSCs (CD90^+^-cells) upon cocultivation with the MM-cell line MM.1S **(F)** Bar chart showing fold changes of MM.1S proliferation either cultured in direct contact with MSCs, preventing contact by an insert or by using MSC-supernatant after 3 d of cultivation, normalized to cell count of MM.1S monoculture; Data are presented as mean ± SEM; Pearson correlation coefficient was used **(A, C, D)**, significance was calculated using an unpaired t-test **(B)**, a paired t-test **(E)** or two-way ANOVA **(F)**; **p* ≤ 0.05, ***p* ≤ 0.01, ****p* ≤ 0.001

Supplementary Figure 3
**(A)** Volcano plot highlighting differential expressed genes of PI3K-AKT-mTOR-pathway of all differential expressed genes in MM-Act- compared to CTR-MSC. Statistically significant data (adjusted p < 0.05) are coloured (yellow - enriched/blue - downregulated) and presented with threshold of the absolute value of Log_2_ fold change>0.585. **(B-D)** Enrichment plot of the NOTCH (B)/MYC **(C)**/Inflammatory Response **(D)** Hallmark gene sets in MM-Act-MSCs (n = 6) as compared to CTR-MSCs (n = 5); NES, normalized enrichment score

Supplementary Figure 4
**(A, B)** Graph, showing the dose-related proliferation changes of MSCs **(A)** and MM-cells **(B)** after 3 d of DMSO or Pictilisib treatment expressed as fold change; normalized to proliferation under DMSO treatment

## Abbreviations

BM, bone marrow; CTR-MSCs, mesenchymal stem cells isolated from patients with other malignant and non-malignant diseases; MM, multiple myeloma; MM-Act-MSCs, mesenchymal stem cells isolated from multiple myeloma patients with active disease; MM-Rm-MSCs, mesenchymal stem cells isolated from multiple myeloma patients in remission; MSCs, mesenchymal stem cells
