# Supplementary figures and images for: Inhibiting PI3K–AKT–mTOR Signaling in Multiple Myeloma-Associated Mesenchymal Stem Cells Impedes the Proliferation of Multiple Myeloma Cells
Source: Front Oncol. 2022 Jun 20;12:874325. doi: 10.3389/fonc.2022.874325 (PMC9251191; doi:10.3389/fonc.2022.874325)

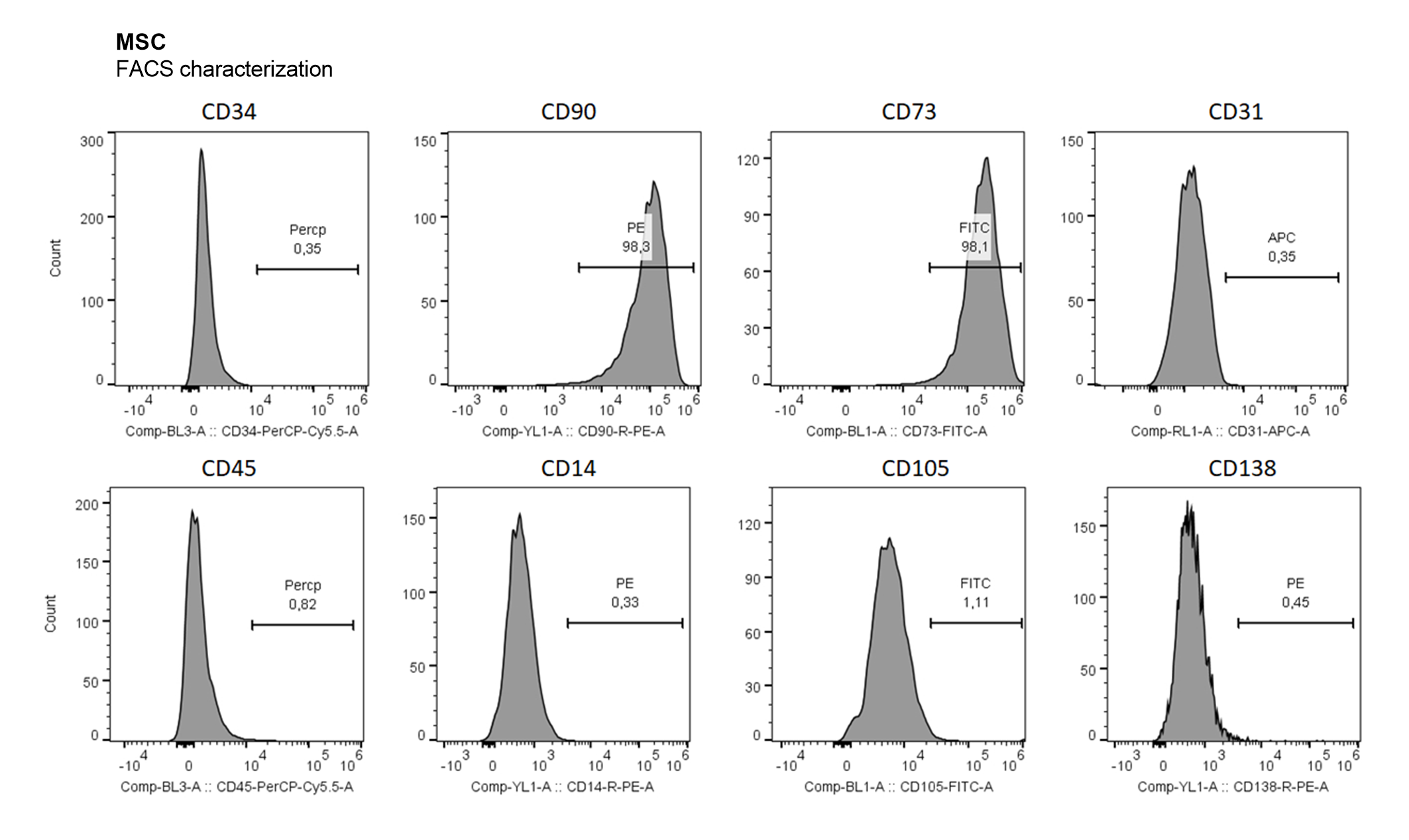

Supplement: Supplementary Figure 1 — Histogram showing an exemplary flow cytometric staining of MM-Act-MSCs in passage 4, positive for the typical MSC-surface markers as CD90, CD73, and weakly positive (in accordance with manufacturer´s instructions) for CD105 and negative for CD14, CD45, CD34, CD31 and CD138 [file Image_1.tif]

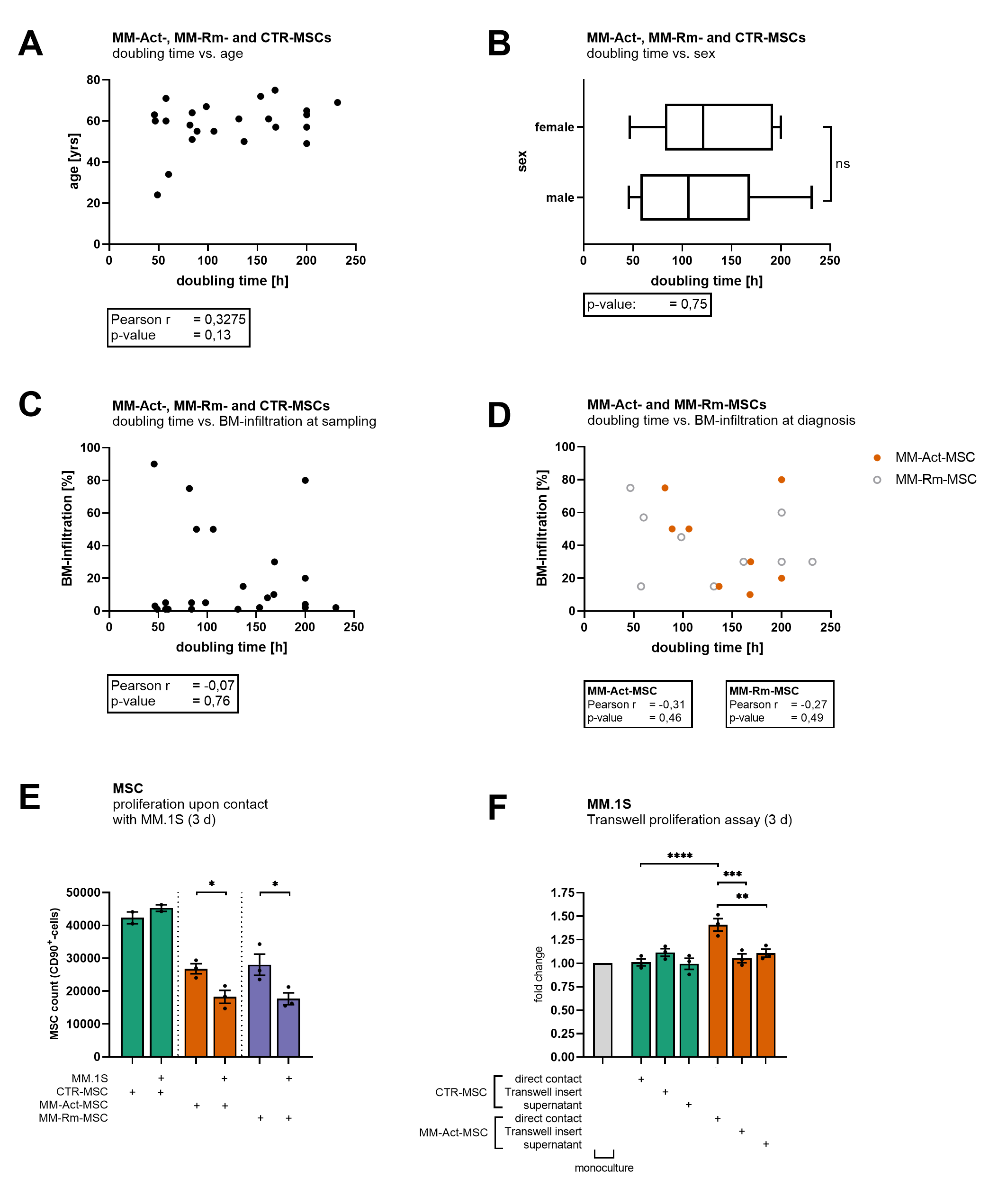

Supplement: Supplementary Figure 2 — (A) Dot plot/Correlative analysis of MSCs´ doubling time vs. donors´ age at sampling (B) Box plot/Comparison of MSCs´ doubling time and donors´ sex. (C, D) Dot plot/Correlative analysis of MSCs´ doubling time vs. BM-infiltration in different subgroups (E) Bar chart showing the count of MSCs (CD90+-cells) upon cocultivation with the MM-cell line MM.1S (F) Bar chart showing fold changes of MM.1S proliferation either cultured in direct contact with MSCs, preventing contact by an insert or by using MSC-supernatant after 3 d of cultivation, normalized to cell count of MM.1S monoculture; Data are presented as mean ± SEM; Pearson correlation coefficient was used (A, C, D), significance was calculated using an unpaired t-test (B), a paired t-test (E) or two-way ANOVA (F); *p ≤ 0.05, **p ≤ 0.01, ***p ≤ 0.001 [file Image_2.tif]

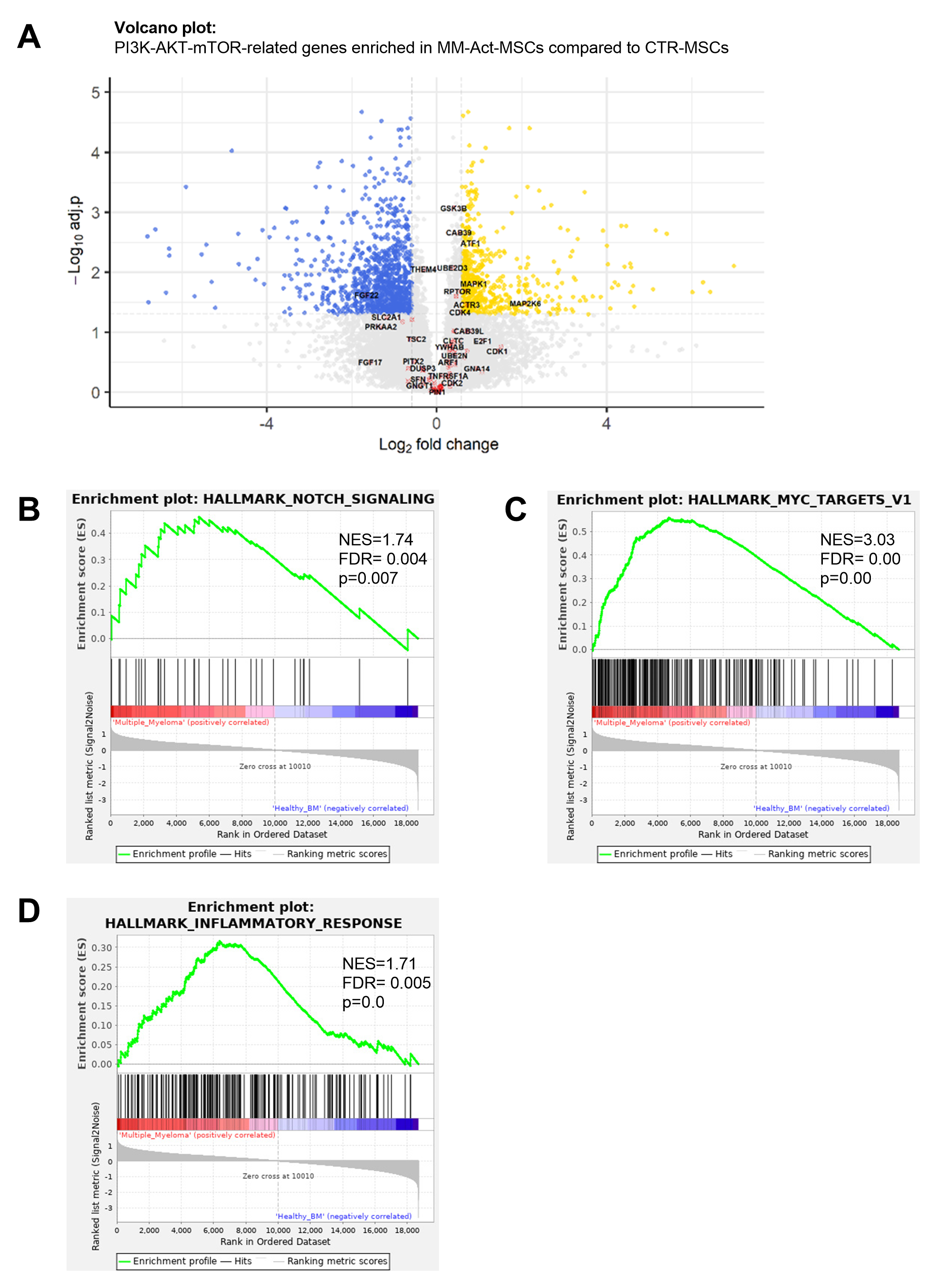

Supplement: Supplementary Figure 3 — (A) Volcano plot highlighting differential expressed genes of PI3K-AKT-mTOR-pathway of all differential expressed genes in MM-Act- compared to CTR-MSC. Statistically significant data (adjusted p < 0.05) are coloured (yellow - enriched/blue - downregulated) and presented with threshold of the absolute value of Log2 fold change>0.585. (B-D) Enrichment plot of the NOTCH (B)/MYC (C)/Inflammatory Response (D) Hallmark gene sets in MM-Act-MSCs (n = 6) as compared to CTR-MSCs (n = 5); NES, normalized enrichment score [file Image_3.tif]

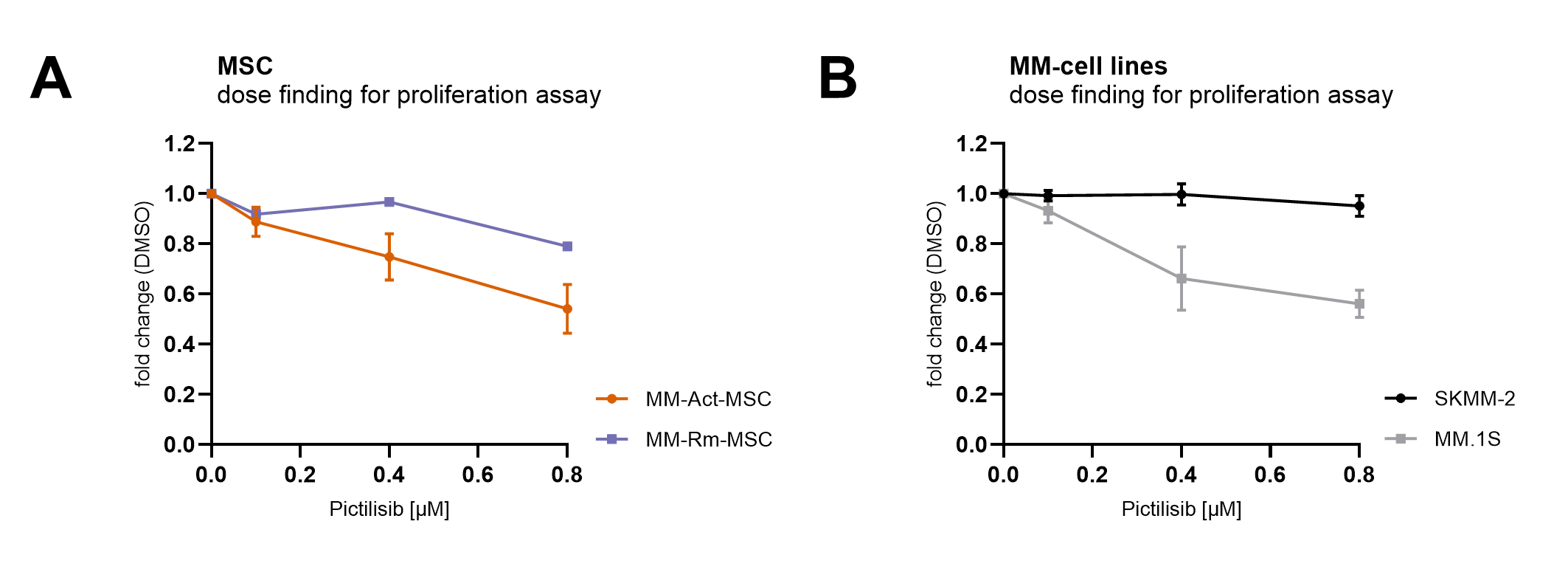

Supplement: Supplementary Figure 4 — (A, B) Graph, showing the dose-related proliferation changes of MSCs (A) and MM-cells (B) after 3 d of DMSO or Pictilisib treatment expressed as fold change; normalized to proliferation under DMSO treatment [file Image_4.tif]
